# Relationships between hypoxia markers and the leptin system, estrogen receptors in human primary and metastatic breast cancer: effects of preoperative chemotherapy

**DOI:** 10.1186/1471-2407-10-320

**Published:** 2010-06-22

**Authors:** Mariusz Koda, Luiza Kanczuga-Koda, Mariola Sulkowska, Eva Surmacz, Stanislaw Sulkowski

**Affiliations:** 1 Department of Pathology, Medical University of Bialystok, Bialystok, Poland; 2 Sbarro Institute for Cancer Research and Molecular Medicine, College of Science and Technology, Temple University, Philadelphia, USA

## Abstract

**Background:**

Tumor hypoxia is marked by enhanced expression of hypoxia-inducible factor-α (HIF-1α) and glucose transporter-1 (Glut-1). Hypoxic conditions have also been associated with overexpression of angiogenic factors, such as leptin. The aim of our study was to analyze the relationships between hypoxia markers HIF-1α, Glut-1, leptin, leptin receptor (ObR) and other breast cancer biomarkers in primary and metastatic breast cancer in patients treated or untreated with preoperative chemotherapy.

**Methods:**

The expression of different biomarkers was examined by immunohistochemistry in 116 primary breast cancers and 65 lymph node metastases. Forty five of these samples were obtained form patients who received preoperative chemotherapy and 71 from untreated patients.

**Results:**

In primary tumors without preoperative chemotherapy, HIF-1α and Glut-1 were positively correlated (p = 0.02, r = 0.437). HIF-1α in primary and metastatic tumors without preoperative therapy positively correlated with leptin (p < 0.0001, r = 0.532; p = 0.013, r = 0.533, respectively) and ObR (p = 0.002, r = 0.319; p = 0.083, r = 0.387, respectively). Hypoxia markers HIF-1α and Glut-1 were negatively associated with estrogen receptor alpha (ERα) and positively correlated with estrogen receptor beta (ERβ). In this group of tumors, a positive correlation between Glut-1 and proliferation marker Ki-67 (p = 0.017, r = 0.433) was noted. The associations between HIF-1α and Glut-1, HIF-1α and leptin, HIF-1α and ERα as well as Glut-1 and ERβ were lost following preoperative chemotherapy.

**Conclusions:**

Intratumoral hypoxia in breast cancer is marked by coordinated expression of such markers as HIF-1α, Glut-1, leptin and ObR. The relationships among these proteins can be altered by preoperative chemotherapy.

## Background

During breast cancer development and progression, rapidly proliferating neoplastic cells are often exposed to hypoxia resulting from insufficient local supply of oxygen and nutrition [1]. Hypoxia can affect developing tumor two-way. On one hand, it might inhibit proliferation and stimulate apoptotic or necrotic cell death, while it can also induce progression of neoplasms and cause resistance to anti-cancer treatments. In developing tumors, hypoxic cells are more resistant to ionizing radiation and chemotherapy, display a more invasive and metastatic phenotype, and increased genetic instability. Limited oxygen concentration in tumor tissue is known to modulate (inhibit or stimulate) gene expression as well as affect metabolic processes [2-5].

Hypoxia-inducible factor-1 (HIF-1) and glucose transporter-1 (Glut-1) are two known markers of tissue oxygenation. HIF-1 is heterodimeric transcription factor that consists of a constitutively expressed HIF-1β subunit and a hypoxia-induced HIF-1α subunit [6,7]. Under normoxic conditions, HIF-1α is rapidly degraded, but hypoxia or activation of certain intracellular pathways, such as the PI-3K and ERK1/2 pathways, induce accumulation of this protein and its translocation to the nucleus. Additionally, HIF-1α expression can be upregulated due to loss of tumor suppressor genes PTEN and p53 [7,8]. HIF-1α binds to Hypoxia Response Elements (HRE, 5'-RCGTG-3') within regulatory regions of target genes whose protein products are implicated in metabolism, growth, angiogenesis and apoptosis [9-11]. Expression of HIF-1α is characteristic of early stages of carcinogenesis and has been correlated with increased intratumoral angiogenesis, cancer progression, poor patient prognosis and chemo- as well as radio- resistance [12-14].

Glucose transporter-1 (Glut-1) is another protein whose expression in tumor is hypoxia-dependent [15-17]. Glut-1 exerts cytoprotective effect enabling glucose transport into hypoxic cells, and preventing hypoxia-induced cell death. Glut-1 accumulation was observed in cancer cells in regions near necrotic areas in tumors [18]. Under hypoxic conditions, Glut-1 expression can be enhanced by HIF-1α [19]. Hayashi *et al*. (2004) found that this phenomenon involves HIF-1α interaction with a HRE site in the Glut-1 promoter [20].

In breast cancer, increased expression of Glut-1 was associated with higher invasiveness of breast cancer cell lines and with the poorly differentiated phenotype in human ductal mammary cancers [21]. Glut-1 overexpression in colorectal cancer cells was associated with rapid cancer progression and inversely correlated with prognosis [22]. In breast and lung cancer cell models, blocking of Glut-1 with specific antibodies reduced growth and induced apoptosis [23]. We found significant coexpression of Glut-1, and apoptotic regulators Bcl-xL and Bax in colorectal cancer, which could suggest cooperation among these proteins in processes such as elimination of cells due to irreversible injury, adaptation to hypoxia, reduction of further damage, and survival [16].

Hypoxia can also upregulate the expression of a cytokine leptin (Ob) - a multifunctional peptide hormone that has been implicated in cancer cell growth, transformation, metastasis and resistance to cancer treatments [24-33]. Our recent study indicated that leptin and the leptin receptor (ObR) were significantly overexpressed in primary breast cancer and lymph node metastasis relative to non-cancer mammary epithelium [34]. The leptin gene promoter contains eight HRE with the minimal core sequence 5'-RCGTG-3' that can recruit HIF-1α [35]. Recently, we demonstrated that in breast cancer cells, leptin and ObR expression can be activated in response to hypoxia or hyperinsulinemia [34,36]. In the case of leptin expression, the process was mediated through HIF-1 and/or Sp-1-dependent transcription [37,38].

Here we analyzed the relationships between hypoxia-related proteins (HIF-1α, Glut-1, leptin, ObR) and other cancer biomarkers in primary breast cancer and lymph node metastases. We also assessed the impact of preoperative chemotherapy (neoadjuvant therapy) on the correlative expression of these proteins.

## Methods

### Patients and tissue samples

The expression of HIF-1α, Glut-1, leptin, ObR, estrogen receptors ERα, ERβ and Ki-67 was assessed in breast cancer samples obtained from 116 women, aged 30-82 years (mean age 54.4), who underwent partial or total mastectomy and lymph node dissection for primary breast cancer. Forty five patients from this group underwent preoperative chemotherapy (neoadjuvant therapy) following Ansfield (cyclophosphamid, prednison, 5-fluorouracyl, metotrexate and vincristin) or CMF (cyclophosphamid, metotrexat, 5-fluorouracyl) regimens. As the aim of the present study was not the evaluation of the impact of a particular preoperative chemotherapy regimen, all the Ansfiled and CMF patients were analyzed as one group.

Tissue samples were fixed in 10% buffered formaldehyde solution, embedded in paraffin blocks at 56°C and stained with hematoxylin-eosin. Histopathological examination of sections was based on the WHO and pTN classification of breast tumors [39]. These human studies were performed in agreement with the ethical standards laid down in the 1964 Declaration of Helsinki and its latest revision in 2000, and approved by the Ethics Committee at Medical University of Bialystok. All subjects signed informed consent before inclusion in the study. In order to uniform the examined group without neoadjuvant therapy, the analysis included selected cases of invasive ductal carcinomas in grade G2 (49 patients without preoperative chemotherapy) and G3 (22 patients without preoperative chemotherapy) and in stage pT1 (43 patients without preoperative chemotherapy) and pT2 (28 patients without preoperative chemotherapy). Tumor stage and grade were impossible to determine in the group of tumors after chemotherapy because of numerous morphological changes in epithelial and stromal tissue components. The presence of lymph node metastases was found at the time of diagnosis in 35 patients (49.3%) who did not receive preoperative chemotherapy and in 30 patients (66.7%) who underwent preoperative chemotherapy.

### Immunohistochemistry

The immunohistochemical analysis of HIF-1α, Glut-1, leptin (Ob), ObR, ERα, ERβ, and Ki-67 expression was carried out using 5 μm consecutive tissue sections obtained from tissue samples, as described by us previously in detail [16,28-30,34,40]. The sections were dewaxed in xylene and rehydrated in graded alcohols. After antigen unmasking and endogenous peroxidase removal, nonspecific binding was blocked by incubating the slides for 1 h with 1.5% normal serum in PBS. Next, the sections were incubated with the primary antibodies (Abs). The following Abs were used for marker detection: for HIF-1α, rabbit polyclonal Ab (sc-10790; Santa Cruz Biotechnology, Santa Cruz, USA), dilution 1:400; for Glut-1, rabbit polyclonal Ab (A3536; Dako, Denmark), dilution 1:250; for leptin, rabbit polyclonal Ab (pAb) A-20 (Santa Cruz, USA), dilution 1:100; for ObR, rabbit pAb H-300 (Santa Cruz, USA), dilution 1:75; for ERα, mouse monoclonal Ab (mAb) F-10 (Santa Cruz, USA), dilution 1:200; for ERβ, rabbit pAb H-150 (Santa Cruz, USA), dilution 1:200; and for Ki-67, mouse mAb MIB-1 (Dako, Denmark), dilution 1:100. The analysis of leptin, ObR, ERα and ERβ was performed with avidin-biotin-peroxidase complex (ABC Staining System, Santa Cruz, USA), for HIF-1α and Glut-1 with EnVision method (Dako, Denmark), and for Ki-67 with streptavidin-biotin-peroxidase complex (LSAB kit, Dako, Denmark) to reveal Ab-antigen reactions. All slides were counterstained with hematoxylin. Breast specimens previously classified as positive for the expression of the studied markers were used for control and protocol standardization. In negative controls, primary Abs were omitted. The expression of leptin, ObR, ERα, ERβ, and Ki-67 was analyzed by light microscopy in 10 different section fields, and the mean percentage of tumor cells displaying positive staining was scored. The expression of leptin and ObR in cancer samples was classified using a four-point scale: 0, <10% positive cells; 1+, 10-50% positive cells with weak staining; 2+, >50% positive cells with weak staining; 3+, >50% positive cells with strong staining. ERα and ERβ were classified as follows: 0, <10% cells with positive staining; 1+, 10-50% cells with positive staining; 2+, 50-80% cells with positive staining; 3+ >80% cells with positive staining. Ki-67 expression was classified as follows: 0, <10% cells with positive staining; 1+, 10-40% cells with positive staining; 2+ >40% cells with positive staining. For HIF-1α and Glut-1, we applied a 3-grade scoring system as follows: 0, <10% immunoreactive cancer cells; 1+, 10-50% immunoreactive cancer cells; 2+, >50% of immunoreactive malignant cells.

### Statistical analysis

Spearman test was used to analyze correlations among leptin, ObR, HIF-1α, Glut-1, ERα, ERβ, and Ki-67 expression in primary and metastatic breast cancer in the group with and without preoperative chemotherapy. Values of p < 0.05 were taken as statistically significant.

## Results

The positivity scores for HIF-1α, Glut-1, leptin, ObR, ERα, ERβ and Ki-67 in primary and metastatic breast cancer in the group without and with preoperative chemotherapy are included in Table 1 and 2. We found a significant correlation between HIF-1α and leptin expression in primary tumors and lymph node metastases in patients who did not receive preoperative chemotherapy (Table 3 and 4). This relationship was not observed in breast tumors of patients who received neoadjuvant therapy. On the other hand, the expression of HIF-1α was correlated with the presence of ObR in primary breast cancer regardless of preoperative treatment (Table 3 and 4). In lymph node metastases from women without neoadjuvant therapy, a trend toward a positive correlation between HIF-1α and ObR was detected (Table 4). However, in lymph node metastases from patients after neodjuvant therapy, HIF-1α and ObR were not associated (Table 4).

**Table 1:** Positivity scores for different biomarkers in primary tumors and lymph node metastases in patients without preoperative chemotherapy.

Biomarkers	Primary tumors; n = 71				Lymph node metastases; n = 35			
	
	0	1+	2+	3+	0	1+	2+	3+
**HIF-1α**	27	21	23	-	11	9	15	-

**GLUT-1**	38	21	12	-	24	5	6	-

**Ob**	10	24	28	9	3	6	15	11

**ObR**	44	16	7	4	17	7	8	3

**ERα**	21	11	27	12	11	8	7	9

**ERβ**	10	22	27	12	5	10	9	11

**Ki-67**	20	29	22	-	9	18	8	-

n - number of studied cases.

**Table 2:** Positivity scores for different biomarkers in primary tumors and lymph node metastases in patients after preoperative chemotherapy.

Biomarkers	Primary tumors; n = 45				Lymph node metastases; n = 30			
	
	0	1+	2+	3+	0	1+	2+	3+
**HIF-1α**	10	24	11	-	5	13	12	-

**GLUT-1**	24	16	5	-	15	11	4	-

**Ob**	9	15	18	3	4	9	14	3

**ObR**	29	11	5	0	12	6	8	4

**ERα**	17	10	11	7	10	6	6	8

**ERβ**	9	9	23	4	5	5	13	7

**Ki-67**	17	20	8	-	11	13	6	-

n - number of studied cases.

**Table 3:** Correlation between HIF-1α, GLUT-1 and other studied biomarkers in primary breast cancer in patients without and after preoperative chemotherapy.

Compared biomarkers	Primary tumors without preoperative chemotherapy; n = 71		Primary tumors after preoperative chemotherapy; n = 45	
	
	p	r	P	r
**HIF-1α - Ob**	**<0.0001**	0.532	0.116	0.288

**HIF-1α - ObR**	**0.002**	0.319	**0.007**	0.445

**HIF-1α - ERα**	**0.055**	-0.194	0.244	0.191

**HIF-1α - ERβ**	**0.002**	0.408	**0.031**	0.346

**HIF-1α - Ki-67**	0.822	-0.031	0.801	0.042

**HIF-1α -GLUT-1**	**0.02**	0.437	0.625	-0.087

**GLUT-1 - Ob**	0.930	0.018	0.431	0.152

**GLUT-1 - ObR**	0.472	0.139	0.338	-0.165

**GLUT-1 - ERα**	**0.022**	-0.410	**0.038**	-0.337

**GLUT-1 - ERβ**	**0.008**	0.467	0.281	0.179

**GLUT-1 - Ki-67**	**0.017**	0.433	**0.050**	0.320

n - number of studied cases.

**Table 4:** Correlation between HIF-1α, GLUT-1 and other studied biomarkers in lymph node metastases in patients without and after preoperative chemotherapy.

Compared biomarkers	Lymph node metastases without preoperative chemotherapy; n = 35		Lymph node metastases after preoperative chemotherapy; n = 30	
	
	P	r	p	r
**HIF-1α - Ob**	**0.013**	0.533	0.465	0.168

**HIF-1α - ObR**	**0.083**	0.387	0.150	0.302

**HIF-1α - ERα**	0.133	-0.327	0.908	0.024

**HIF-1α - ERβ**	0.119	0.474	0.188	0.272

**HIF-1α - Ki-67**	0.604	-0.167	0.951	-0.013

**HIF-1α -GLUT-1**	0.776	-0.177	0.401	-0.198

**GLUT-1 - Ob**	0.405	0.242	0.361	-0.209

**GLUT-1 - ObR**	0.261	-0.309	0.335	-0.216

**GLUT-1 - ERα**	**0.017**	-0.606	**0.021**	-0.469

**GLUT-1 - ERβ**	0.677	-0.117	0.785	0.059

**GLUT-1 - Ki-67**	**0.078**	0.468	0.749	0.069

n - number of studied cases.

Glut-1 did not correlate with leptin or ObR in any studied group of breast tumors (Table 3 and 4). On the other hand, a positive correlation between HIF-1α and Glut-1 was found in primary tumors, but not in lymph node metastasis in patients without preoperative chemotherapy (Table 3). The association between HIF-1α and Glut-1 was not observed in primary or metastatic cancers in patients who received neoadjuvant therapy (Table 3 and 4).

Next, we studied relationships between hypoxia markers and estrogen receptor expression. In primary tumors without preoperative chemotherapy, we noted a trend toward a negative correlation between HIF-1α expression and ERα (p = 0.055, r= -0.194; Table 3). These associations were absent in lymph node metastases. HIF-1α and ERα did not correlate in primary tumors and metastases from patients who underwent preoperative chemotherapy (Table 3 and 4).

Glut-1 negatively correlated with ERα expression in primary tumors and in lymph node metastases in biopsies from all patients, regardless of preoperative treatment (Table 3 and 4). ERβ positively correlated with HIF-1α in primary tumors, both in patients with and without preoperative chemotherapy (Table 3), and with Glut-1 in primary tumors without preoperative chemotherapy (Table 3).

Finally, we evaluated the link between HIF-1α, Glut-1 and the proliferation marker Ki-67. A positive correlation between Glut-1 and Ki-67 was noted in primary and metastatic cancer biopsies from patients without preoperative chemotherapy (Table 3, 4). In the group of primary tumors after neodjuvant therapy, we observed a trend toward a positive correlation in primary tumors only (Table 3). On the other hand, HIF-1α did not correlate with Ki-67 in primary tumors or in lymph node metastases in both studied groups of patients (Table 3 and 4).

## Discussion

In this study we analyzed the relationships among hypoxia-inducible proteins (i.e., HIF-1α, Glut-1, leptin, and ObR) and other biomarkers (i.e., estrogen receptors and Ki-67) in primary and metastatic breast cancer. Our second goal was to evaluate potential influence of preoperative chemotherapy on associations among studied proteins. The major findings of our study can be summarized as follows: 1) HIF-1α and Glut-1 are positively correlated in primary tumors without preoperative chemotherapy; 2) HIF-1α positively correlate with the leptin system in primary and metastatic breast cancer without preoperative chemotherapy; 3) the leptin system is not associated with Glut-1 expression in all studied groups; 4) Glut-1 correlates negatively with ERα in primary and metastatic tumors, regardless of preoperative chemotherapy; 5) hypoxia markers correlate positively with ERβ expression in primary tumors, especially in the group without preoperative therapy; 6) Glut-1 expression is positively associated with Ki-67 in primary tumors, while in lymph node metastases, a trend toward positive correlation between these proteins is found in the group without therapy; 7) preoperative chemotherapy influences the associations between HIF-1α and leptin in primary and metastatic tumors, HIF-1α and ObR in metastatic tumors, HIF-1α and Glut-1 in primary tumors; Glut-1 and ERβ in primary tumors; Glut-1 and Ki-67 in primary and metastatic tumors.

Hypoxia in solid tumors is associated with the accumulation of HIF-1α and activation of HIF-1-dependent transcription of genes regulating cell motility, invasion, and angiogenesis [14,41]. Bos *et al*. (2001, 2003) reported that HIF-1α overexpression was associated with more aggressive breast cancer. We speculate that HIF-1 could also improve glucose consumption in hypoxic cancer cells by the stimulation of glucose transporter Glut-1 expression [12,42]. Our results suggest that hypoxic conditions resulted in coordinated upregulation of HIF-1α and Glut-1 in primary tumors. It is consistent with results of Li *et al*. (2006) who showed that knockdown of HIF-1α in MCF-7 breast cancer cells attenuated the expression of Glut-1 mRNA and other genes [43]. Additionally, the HIF-1α knockdown cells displayed increased sensitivity to chemotherapeutic agents [43]. Interestingly, we noted that the association between HIF-1α and Glut-1 was lost in lymph node metastases as well as in tumors after preoperative chemotherapy. Potentially, this could be related to downregulation of HIF-1 coregulators, such as p300 [44], due to neoadjuvant therapy or genetic rearrangements in metastatic cells [45], which could result in deficient Glut-1 gene transcription [46,47].

The associations between HIF-1α and the leptin system identified by us in this study confirm the results obtained by Cascio *et al*. (2008), Bartella *et al*. (2008) and Garofalo *et al*. (2006) who demonstrated that physiologic hypoxia and/or accumulation of HIF-1α due to hypoxia-mimicking conditions or stabilization of HIF-1a by growth factors can stimulate leptin and/or ObR expression in breast cancer cells [34,36,37].

According to our knowledge, the present analysis is the first to reveal a positive correlation between HIF-1α and leptin, as well as between HIF-1α and ObR not only in primary tumors, but also in metastasis [31,33,34,36-38]. We reported analogous associations in human colorectal cancer [30]. Cumulatively, present and previous data suggest the involvement of tissue hypoxia in the stimulation of leptin and ObR expression in human cancers [28,30,34]. The overexpression of the leptin system could lead to leptin-enhanced tumor growth and progression under hypoxic conditions.

One of the features of breast cancer progression is the development of resistance to hormonal therapy. The possible mechanism of this phenomenon could be related to differential ER expression in metastatic and primary sites, as described by us before [40]. Our present study suggests that tumor malignancy might correlate with loss of ERα expression. Indeed, hypoxia could induce ERα downregulation via proteasome-dependent pathway [48,49]. Cho *et al*. (2005) suggested that ERα downregulation under hypoxic conditions in human breast cancer involves protein interactions between ERα and HIF-1α [50].

Negative correlation between Glut-1 and ERα found in this study suggests that loss of ERα in breast cancer is associated with overexpression of Glut-1, which could facilitate proliferation, survival and possibly progression and dedifferentiation of cancer cells [51,52].

In contrast to ERα, we noted a positive correlation between ERβ and hypoxia markers HIF-1α, Glut-1. Our observations are consistent with results of Cordadini *et al*. (2004), who reported that hypoxic conditions upregulated ERβ protein levels in breast cancer cells [53]. Moreover, sequence analysis of the ERβ promoter region contains specific sequence for HRE, which might explain coexpression of ERβ and HIF-1α.

Although preoperative chemotherapy is an integral part of management of patients with advanced breast cancer, its impact on cancer biomarkers and their interrelationships in primary and metastatic tumors is poorly recognized. Our study clearly suggest that preoperative chemotherapy alters the expression of and relationships among several hypoxia-related proteins, specifically, HIF-1α and leptin in primary and metastatic tumors, HIF-1α and ObR in metastatic tumors, HIF-1α and Glut-1 in primary tumors; Glut-1 and ERβ in primary tumors; Glut-1 and Ki-67 in primary and metastatic tumors. These data are reminiscent of previous observations pointing to differential expression of ERα, ERβ and Ki-67 expression in metastatic breast cancer versus primary cancer, and significant influence of preoperative chemotherapy on these biomarkers [40,43,54-56].

## Conclusions

In conclusion, our results suggest that breast tumor cells experience hypoxic conditions, as indicated by the expression of such markers as HIF-1α, Glut-1, leptin and ObR. Interestingly, the relationships among these proteins differ in primary and metastatic tumors and might be influenced by preoperative chemotherapy. Thus, analysis of biomarkers in both primary and metastatic tumors before and after chemotherapy could help in understanding the mechanisms of breast cancer progression and select the optimal individualized treatment options.

## Competing interests

The authors declare that they have no competing interests.

## Authors' contributions

MK designed the study, analyzed data and drafted the manuscript; LKK performed statistical analysis and edited the manuscript; MS collected the samples, performed pathological evaluation and edited the manuscript; ES analyzed results and edited the manuscript; SS designed the study, collected the samples, performed pathological evaluation and edited the manuscript.

## Pre-publication history

The pre-publication history for this paper can be accessed here:

http://www.biomedcentral.com/1471-2407/10/320/prepub

## References

[B1] Vaupel P Mayer A Hypoxia in cancer: significance and impact on clinical outcome Cancer Metastasis Rev 2007 26 225 239 10.1007/s10555-007-9055-1 17440684

[B2] Liao D Corle C Seagroves TN Johnson RS Hypoxia-inducible factor-1alpha is a key regulator of metastasis in a transgenic model of cancer initiation and progression Cancer Res 2007 67 563 572 10.1158/0008-5472.CAN-06-2701 17234764

[B3] Van den Eynden GG Van der Auwera I Van Laere SJ Colpaert CG Turley H Harris AL van Dam P Dirix LY Vermeulen PB Van Marck EA Angiogenesis and hypoxia in lymph node metastases is predicted by the angiogenesis and hypoxia in the primary tumour in patients with breast cancer Br J Cancer 2005 93 1128 1136 10.1038/sj.bjc.6602828 16251878 PMC2361504

[B4] Vaupel P Mayer A Briest S Höckel M Hypoxia in breast cancer: role of blood flow, oxygen diffusion distances, and anemia in the development of oxygen depletion Adv Exp Med Biol 2005 566 333 342 full_text 16594170 10.1007/0-387-26206-7_44

[B5] Gardner LB Corn PG Hypoxic regulation of mRNA expression Cell Cycle 2008 7 1916 1924 18604161 10.4161/cc.7.13.6203

[B6] Wang GL Semenza GL Purification and characterization of hypoxia-inducible factor 1 J Biol Chem 1995 270 1230 1237 10.1074/jbc.270.3.1230 7836384

[B7] Wang GL Jiang BH Rue EA Semenza GL Hypoxia-inducible factor 1 is a basic-helix-loop-helix-PAS heterodimer regulated by cellular O2 tension Proc Natl Acad Sci USA 1995 92 5510 5514 10.1073/pnas.92.12.5510 7539918 PMC41725

[B8] Zundel W Schindler C Haas-Kogan D Koong A Kaper F Chen E Gottschalk AR Ryan HE Johnson RS Jefferson AB Stokoe D Giaccia AJ Loss of PTEN facilitates HIF-1-mediated gene expression Genes Dev 2000 14 391 396 10691731 PMC316386

[B9] Semenza GL Targeting HIF-1 for cancer therapy Nat Rev Cancer 2003 3 721 732 10.1038/nrc1187 13130303

[B10] Marignol L Lawler M Coffey M Hollywood D Achieving hypoxia-inducible gene expression in tumors Cancer Biol Ther 2005 4 359 364 15846086 10.4161/cbt.4.4.1646

[B11] Nangaku M Eckardt KU Hypoxia and the HIF system in kidney disease J Mol Med 2007 85 1325 1330 10.1007/s00109-007-0278-y 18026918

[B12] Bos R Zhong H Hanrahan CF Mommers EC Semenza GL Pinedo HM Abeloff MD Simons JW van Diest PJ van der Wall E Levels of hypoxia-inducible factor-1 alpha during breast carcinogenesis J Natl Cancer Inst 2001 93 309 314 10.1093/jnci/93.4.309 11181778

[B13] Generali D Berruti A Brizzi MP Campo L Bonardi S Wigfield S Bersiga A Allevi G Milani M Aguggini S Gandolfi V Dogliotti L Bottini A Harris AL Fox SB Hypoxia-inducible factor-1α expression predicts a poor response to primary chemoendocrine therapy and disease-free survival in primary human breast cancer Clin Cancer Res 2006 12 4562 4568 10.1158/1078-0432.CCR-05-2690 16899602

[B14] Zhong H De Marzo AM Laughner E Lim M Hilton DA Zagzag D Buechler P Isaacs WB Semenza GL Simons JW Overexpression of hypoxia-inducible factor 1α in common human cancers and their metastases Cancer Res 1999 59 5830 5835 10582706

[B15] Gatenby RA Smallbone K Maini PK Rose F Averill J Nagle RB Worrall L Gillies RJ Cellular adaptations to hypoxia and acidosis during somatic evolution of breast cancer Br J Cancer 2007 97 646 653 10.1038/sj.bjc.6603922 17687336 PMC2360372

[B16] Wincewicz A Sulkowska M Koda M Kanczuga-Koda L Witkowska E Sulkowski S Significant coexpression of GLUT-1, Bcl-xL, and Bax in colorectal cancer Ann N Y Acad Sci 2007 1095 53 61 10.1196/annals.1397.007 17404017

[B17] Sulkowska M Wincewicz A Sulkowski S Koda M Kanczuga-Koda L Relations of TGF-β1 with HIF-1α, GLUT-1 and longer survival of colorectal cancer patients Pathology 2009 41 254 260 10.1080/00313020802579318 19142800

[B18] Cooper R Sarioğlu S Sökmen S Füzün M Küpelioğlu A Valentine H Görken IB Airley R West C Glucose transporter-1 (GLUT-1): a potential marker of prognosis in rectal carcinoma? Br J Cancer 2003 89 870 876 10.1038/sj.bjc.6601202 12942120 PMC2394489

[B19] Williams KJ Telfer BA Airley RE Peters HP Sheridan MR van der Kogel AJ Harris AL Stratford IJ A protective role for HIF-1 in response to redox manipulation and glucose deprivation: implications for tumorigenesis Oncogene 2002 21 282 290 10.1038/sj.onc.1205047 11803471

[B20] Hayashi M Sakata M Takeda T Yamamoto T Okamoto Y Sawada K Kimura A Minekawa R Tahara M Tasaka K Murata Y Induction of glucose transporter 1 expression through hypoxia-inducible factor 1α under hypoxic conditions in trophoblast-derived cells J Endocrinol 2004 183 145 154 10.1677/joe.1.05599 15525582

[B21] Grover-McKay M Walsh SA Seftor EA Thomas PA Hendrix MJ Role for glucose transporter 1 protein in human breast cancer Pathol Oncol Res 1998 4 115 120 10.1007/BF02904704 9654596

[B22] Haber RS Rathan A Weiser KR Pritsker A Itzkowitz SH Bodian C Slater G Weiss A Burstein DE GLUT1 glucose transporter expression in colorectal carcinoma: a marker for poor prognosis Cancer 1998 83 34 40 10.1002/(SICI)1097-0142(19980701)83:1<34::AID-CNCR5>3.0.CO;2-E 9655290

[B23] Rastogi S Banerjee S Chellappan S Simon GR Glut-1 antibodies induce growth arrest and apoptosis in human cancer cell lines Cancer Lett 2007 257 244 251 10.1016/j.canlet.2007.07.021 17910902

[B24] Garofalo C Surmacz E Leptin and cancer J Cell Physiol 2006 207 12 22 10.1002/jcp.20472 16110483

[B25] Surmacz E Obesity hormone leptin: a new target in breast cancer? Breast Cancer Res 2007 9 301 17274833 10.1186/bcr1638 PMC1851379

[B26] Riolfi M Ferla R Del Valle L Piña-Oviedo S Scolaro L Micciolo R Guidi M Terrasi M Cetto GL Surmacz E Leptin and its receptor are overexpressed in brain tumors and correlate with the degree of malignancy Brain Pathol in press 19775291 10.1111/j.1750-3639.2009.00323.x PMC2864337

[B27] Sulkowska M Golaszewska J Wincewicz A Koda M Baltaziak M Sulkowski S Leptin - from regulation of fat metabolism to stimulation of breast cancer growth Pathol Oncol Res 2006 12 69 72 10.1007/BF02893446 16799705

[B28] Koda M Sulkowska M Wincewicz A Kanczuga-Koda L Musiatowicz B Szymanska M Sulkowski S Expression of leptin, leptin receptor, and hypoxia-inducible factor 1α in human endometrial cancer Ann N Y Acad Sci 2007 1095 90 98 10.1196/annals.1397.013 17404022

[B29] Koda M Sulkowska M Kanczuga-Koda L Surmacz E Sulkowski S Overexpression of the obesity hormone leptin in human colorectal cancer J Clin Pathol 2007 60 902 906 10.1136/jcp.2006.041004 17660334 PMC1994494

[B30] Koda M Sulkowska M Kanczuga-Koda L Cascio S Colucci G Russo A Surmacz E Sulkowski S Expression of the obesity hormone leptin and its receptor correlates with hypoxia-inducible factor-1α in human colorectal cancer Ann Oncol 2007 18 Suppl 6 116 119 17591803 10.1093/annonc/mdm238

[B31] Koda M Sulkowska M Kanczuga-Koda L Jarzabek K Sulkowski S Expression of leptin and its receptor in female breast cancer in relation with selected apoptotic markers Folia Histochem Cytobiol 2007 45 Suppl 1 187 191 18292831

[B32] Vona-Davis L Rose DP Adipokines as endocrine, paracrine, and autocrine factors in breast cancer risk and progression Endocr Relat Cancer 2007 14 189 206 10.1677/ERC-06-0068 17639037

[B33] Fiorio E Mercanti A Terrasi M Micciolo R Remo A Auriemma A Molino A Parolin V Di Stefano B Bonetti F Giordano A Cetto GL Surmacz E Leptin/HER2 crosstalk in breast cancer: in vitro study and preliminary in vivo analysis BMC Cancer 2008 8 305 10.1186/1471-2407-8-305 18945363 PMC2588622

[B34] Garofalo C Koda M Cascio S Sulkowska M Kanczuga-Koda L Golaszewska J Russo A Sulkowski S Surmacz E Increased expression of leptin and the leptin receptor as a marker of breast cancer progression: possible role of obesity-related stimuli Clin Cancer Res 2006 12 1447 1453 10.1158/1078-0432.CCR-05-1913 16533767

[B35] Grosfeld A Andre J Hauguel-De Mouzon S Berra E Pouyssegur J Guerre-Millo M Hypoxia-inducible factor 1 transactivates the human leptin gene promoter J Biol Chem 2002 277 42953 42957 10.1074/jbc.M206775200 12215445

[B36] Bartella V Cascio S Fiorio E Auriemma A Russo A Surmacz E Insulin-dependent leptin expression in breast cancer cells Cancer Res 2008 68 4919 4927 10.1158/0008-5472.CAN-08-0642 18559540

[B37] Cascio S Bartella V Auriemma A Johannes GJ Russo A Giordano A Surmacz E Mechanism of leptin expression in breast cancer cells: role of hypoxia-inducible factor-1α Oncogene 2008 27 540 547 10.1038/sj.onc.1210660 17653093

[B38] Terrasi M Fiorio E Mercanti A Koda M Moncada CA Sulkowski S Merali S Russo A Surmacz E Functional analysis of the -2548G/A leptin gene polymorphism in breast cancer cells Int J Cancer 2009 125 1038 1044 10.1002/ijc.24372 19408304

[B39] Tavassoli FA Tavassoli FA, Devilee P Tumours of the breast Pathology and genetics of tumours of the breast and female genital organs 2003 Lyon: IARC Press 9 112

[B40] Koda M Sulkowski S Kanczuga-Koda L Surmacz E Sulkowska M Expression of ERα, ERβ and Ki-67 in primary tumors and lymph node metastases in breast cancer Oncol Rep 2004 11 753 759 15010868

[B41] Hirota K Semenza GL Regulation of angiogenesis by hypoxia-inducible factor 1 Crit Rev Oncol Hematol 2006 59 15 26 10.1016/j.critrevonc.2005.12.003 16716598

[B42] Bos R van der Groep P Greijer AE Shvarts A Meijer S Pinedo HM Semenza GL van Diest PJ van der Wall E Levels of hypoxia-inducible factor-1α independently predict prognosis in patients with lymph node negative breast carcinoma Cancer 2003 97 1573 1581 10.1002/cncr.11246 12627523

[B43] Li J Shi M Cao Y Yuan W Pang T Li B Sun Z Chen L Zhao RC Knockdown of hypoxia-inducible factor-1alpha in breast carcinoma MCF-7 cells results in reduced tumor growth and increased sensitivity to methotrexate Biochem Biophys Res Commun 2006 342 1341 1351 10.1016/j.bbrc.2006.02.094 16516853

[B44] Freedman SJ Sun ZY Poy F Kung AL Livingston DM Wagner G Eck MJ Structural basis for recruitment of CBP/p300 by hypoxia-inducible factor-1 alpha Proc Natl Acad Sci USA 2002 99 5367 5372 10.1073/pnas.082117899 11959990 PMC122775

[B45] Zhang C Li K Wei L Li Z Yu P Teng L Wu K Zhu J p300 expression repression by hypermethylation associated with tumour invasion and metastasis in oesophageal squamous cell carcinoma J Clin Pathol 2007 60 1249 1253 10.1136/jcp.2006.044099 17965222 PMC2095476

[B46] Chen C Pore N Behrooz A Ismail-Beigi F Maity A Regulation of glut1 mRNA by hypoxia-inducible factor-1. Interaction between H-ras and hypoxia J Biol Chem 2001 276 9519 9525 10.1074/jbc.M010144200 11120745

[B47] Vleugel MM Shvarts D van der Wall E van Diest PJ p300 and p53 levels determine activation of HIF-1 downstream targets in invasive breast cancer Hum Pathol 2006 37 1085 1092 10.1016/j.humpath.2006.03.015 16867872

[B48] Cooper C Liu GY Niu YL Santos S Murphy LC Watson PH Intermittent hypoxia induces proteasome-dependent down-regulation of estrogen receptor alpha in human breast carcinoma Clin Cancer Res 2004 10 8720 8727 10.1158/1078-0432.CCR-04-1235 15623657

[B49] Kurebayashi J Otsuki T Moriya T Sonoo H Hypoxia reduces hormone responsiveness of human breast cancer cells Jpn J Cancer Res 2001 92 1093 1101 11676860 10.1111/j.1349-7006.2001.tb01064.x PMC5926610

[B50] Cho J Kim D Lee S Lee Y Cobalt chloride-induced estrogen receptor alpha down-regulation involves hypoxia-inducible factor-1α in MCF-7 human breast cancer cells Mol Endocrinol 2005 19 1191 1199 10.1210/me.2004-0162 15695373

[B51] Kang SS Chun YK Hur MH Lee HK Kim YJ Hong SR Lee JH Lee SG Park YK Clinical significance of glucose transporter 1 (GLUT1) expression in human breast carcinoma Jpn J Cancer Res 2002 93 1123 1128 12417042 10.1111/j.1349-7006.2002.tb01214.x PMC5926879

[B52] Rastogi S Banerjee S Chellappan S Simon GR Glut-1 antibodies induce growth arrest and apoptosis in human cancer cell lines Cancer Lett 2007 257 244 251 10.1016/j.canlet.2007.07.021 17910902

[B53] Coradini D Pellizzaro C Speranza A Daidone MG Hypoxia and estrogen receptor profile influence the responsiveness of human breast cancer cells to estradiol and antiestrogens Cell Mol Life Sci 2004 61 76 82 10.1007/s00018-003-3324-0 14704855 PMC11138667

[B54] Koda M Lenczewski A Sulkowska M Kanczuga-Koda L Wincewicz A Tomaszewski J Sulkowski S The effect of chemotherapy on status of estrogen receptors in primary tumors and lymph node metastases of human ductal breast cancer Oncol Rep 2007 17 385 391 17203178

[B55] Koda M Sulkowska M Kanczuga-Koda L Tomaszewski J Kucharczuk W Lesniewicz T Cymek S Sulkowski S The effect of chemotherapy on Ki-67, Bcl-2 and Bak expression in primary tumors and lymph node metastases of breast cancer Oncol Rep 2007 18 113 119 17549355

[B56] Jain V Landry M Levine EA The stability of estrogen and progesterone receptors in patients receiving preoperative chemotherapy for locally advanced breast carcinoma Am Surg 1996 62 162 165 8554195

